# Evidence for DNA methylation mediating genetic liability to non-syndromic cleft lip/palate

**DOI:** 10.2217/epi-2018-0091

**Published:** 2019-01-14

**Authors:** Laurence J Howe, Tom G Richardson, Ryan Arathimos, Lucas Alvizi, Maria R Passos-Bueno, Philip Stanier, Ellen Nohr, Kerstin U Ludwig, Elisabeth Mangold, Michael Knapp, Evie Stergiakouli, Beate St Pourcain, George Davey Smith, Jonathan Sandy, Caroline L Relton, Sarah J Lewis, Gibran Hemani, Gemma C Sharp

**Affiliations:** 1 MRC Integrative Epidemiology Unit, Population Health Sciences, University of Bristol, BS8 2BN, UK; 2 Institute of Cardiovascular Science, University College London, London, NW1 2DA, UK; 3 Centro de Pesquisas Sobre o Genoma Humano eCélulas-Tronco, Instituto de Biociências, Universidade de São Paulo, São Paulo, Brazil; 4 Genetics & Genomic Medicine, UCL Great Ormond Street Institute of Child Health, University College London, London, WC1N 3JH, UK; 5 Institute of Public Health, Aarhus University, Aarhus, Denmark; 6 Institute of Human Genetics, University of Bonn, 53127 Bonn, Germany; 7 Department of Genomics, Life & Brain Center, University of Bonn, 53127 Bonn, Germany; 8 Institute of Medical Biometry, Informatics & Epidemiology, University of Bonn, 53127 Bonn, Germany; 9 Max Planck Institute for Psycholinguistics, Nijmegen, 6525 XD, Netherlands; 10 Donders Institute, 6525 EN Nijmegen, The Netherlands; 11 Bristol Dental School, University of Bristol, BS8 2BN, UK

**Keywords:** ALSPAC, epigenetics, mendelian randomization, nsCL/P

## Abstract

**Aim:**

To determine if nonsyndromic cleft lip with or without cleft palate (nsCL/P) genetic risk variants influence liability to nsCL/P through gene regulation pathways, such as those involving DNA methylation.

**Materials & methods:**

nsCL/P genetic summary data and methylation data from four studies were used in conjunction with Mendelian randomization and joint likelihood mapping to investigate potential mediation of nsCL/P genetic variants.

**Results & conclusion:**

Evidence was found at *VAX1* (10q25.3), *LOC146880* (17q23.3) and *NTN1* (17p13.1), that liability to nsCL/P and variation in DNA methylation might be driven by the same genetic variant, suggesting that genetic variation at these loci may increase liability to nsCL/P by influencing DNA methylation. Follow-up analyses using different tissues and gene expression data provided further insight into possible biological mechanisms.

Orofacial clefts are a heterogeneous group of congenital malformations [1]. In epidemiology and genetics research, orofacial clefts are often divided into the subtypes cleft palate only (CPO) and cleft lip with or without cleft palate (CL/P), with strong evidence for distinct etiologies [1]. There is also accumulating evidence suggesting that the CL/P subtypes cleft lip only (CLO) and cleft lip with cleft palate (CLP) may also differ etiologically [2,3]. Mendelian syndromes can feature CL/P and CPO but around 70% of CL/P cases are non-syndromic (nsCL/P), with a complex etiology likely to involve genetic and environmental risk factors [4,5], in addition to stochastic events which are not driven by germline genetic or environmental exposures as these are commonly understood [6].

Genome-wide association studies (GWAS) have identified around 40 distinct genetic risk variants for nsCL/P in European and Asian populations [2,7–14], but many variants reside in non-protein coding regions and so their functional relevance remains unclear. One possibility is that genetic risk variants may be affecting nsCL/P susceptibility through gene regulation pathways. Indeed, a noncoding interval at 8q24, a major nsCL/P risk locus, has previously been shown to regulate gene expression in the developing murine face, suggesting similar mechanisms in humans [15]. There is increasing evidence that epigenetic mechanisms, such as DNA methylation, play a role in development of orofacial clefts [16–19], potentially via changes to gene expression.

In this study, we applied a recently devised analysis framework [20,21] to explore whether genetic influences on liability to nsCL/P are mediated by DNA methylation, using DNA methylation assayed in whole blood as a proxy for DNA methylation in more relevant tissues. Genetic variants that are associated with DNA methylation (methylation quantitative trait loci; mQTL) have been previously identified in the Avon Longitudinal Study of Parents and Children (ALSPAC) [22]. We used these mQTL to perform Mendelian randomization (MR), an epidemiological tool typically used to explore causal relationships between modifiable risk factors and health or disease outcomes [23]. In this instance, genetic variants robustly associated with DNA methylation were tested for association with nsCL/P. We considered four possible models to explain an association between a mQTL and nsCL/P: DNA methylation mediates the effect of a genetic variant on liability to nsCL/P; the direction of effect is reversed, in other words, an individual's overall genetic liability to nsCL/P causes variation in DNA methylation; DNA methylation and liability to nsCL/P are influenced by separate genetic variants that are in linkage disequilibrium (LD) with each other; DNA methylation and liability to nsCL/P are influenced by the same genetic variant, but via independent pathways, in other words, the association is due to horizontal pleiotropy (Figure 1) [20,21].

**Figure 1.**
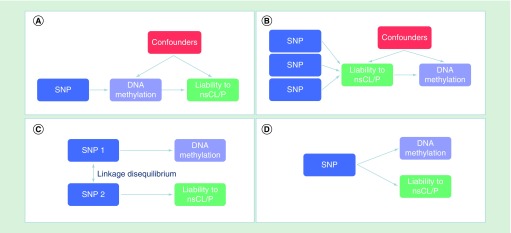
**Possible explanations for an association between methylation quantitative trait loci and nonsyndromic cleft lip with or without cleft palate.** These include **(A)** mediation, **(B)** reverse causation, **(C)** linkage and **(D)** horizontal pleiotropy. In this study, we attempted to identify loci where genetic influences on nonsyndromic cleft lip with or without cleft palate are mediated by DNA methylation, i.e. (A). nsCL/P: Nonsyndromic cleft lip with or without palate; SNP: Single nucleotide polymorphism.

We systematically applied additional analyses, including bidirectional MR and colocalization, to estimate the most likely model as far as possible, although we were unable to use recently derived MR methods [24,25] to distinguish between mediation and horizontal pleiotropy because most CpGs are instrumented by a single genetic variant. Where there was evidence that genetic influences on liability to nsCL/P may be mediated by DNA methylation, we explored associations with gene expression. We also compared our findings relating to genetic liability to nsCL/P in the general population to results from an epigenome-wide association study (EWAS) of whole blood samples from nsCL/P cases and unaffected controls. Given accumulating evidence that different subtypes of orofacial clefts have distinct etiologies, we also explored whether identified CpGs are differentially methylated in blood samples from children with different orofacial cleft subtypes. Finally, since the majority of our analyses used DNA methylation derived in blood, which might not be representative of the developing orofacial tissues, as described previously [16], we explored correlations between DNA methylation in blood and lip/palate tissue in the same individuals, under the (potentially false) assumption that methylation signatures in these tissues might reflect those of the developing orofacial tissues more accurately.

## Methods

### Data sources

#### nsCL/P genetic risk variants

We identified single nucleotide polymorphisms (SNPs) associated with nsCL/P in European populations by conducting a meta-analysis of summary statistics from two nsCL/P GWAS. Summary statistics for the first GWAS came from a case–control study of 399 cases and 1,318 controls of central European descent [8]. For the second GWAS, we generated summary statistics by conducting a GWAS using individual level data from 638 parent-offspring trios and 178 parent-offspring duos of European descent from the International Consortium to Identify Genes and Interactions Controlling Oral Clefts (ICC). These data were available to download from dbGaP (Study accession phs000094.v1.p1) [26]. Full GWAS methods are described in the Supplementary Material, but briefly, we performed a transmission disequilibrium test [27] on the pedigree data. We then performed a fixed-effects inverse-variance-weighted meta-analysis of the summary statistics from both GWAS using METAL [28] on the total sample of 1215 cases and 2772 controls. The results compared well with those previously published using a very similar dataset but slightly different quality control and analysis methods [7]. We used LiftOver (genome.sph.umich.edu/wiki/LiftOver) to convert the genome positions in the nsCL/P summary statistics to the most recent genome build 37. Finally, we used PLINK [29] and data from the ALSPAC as a reference panel to clump the results according to LD (r^2^ < 0.001), within a 250 kb region around each index variant, and generate a set of independent SNPs for the pipeline.

#### Methylation genetic risk variants (mQTL)

##### ALSPAC

To identify mQTL (SNPs associated with DNA methylation), we used data from ALSPAC [30,31]. In addition to collecting detailed questionnaire and clinic data for the whole cohort, the study has generated genome-wide DNA methylation and genotype data for subsets of the cohort [32] (methods described in the Supplementary Material). These data have previously been used to generate a database of mQTL [22]. The database contains summary statistics for all mQTL with a p-value < 1 × 10^-7^ for the association between SNP and CpG. For the purposes of this study, we focused on the mQTL identified in cord blood samples collected at birth (the closest available time point to the orofacial developmental period). For part of our study (the reverse two sample MR), we required specific associations that were unavailable from mQTLdb.org. Therefore, for required CpGs, we replicated the methods in the original study: we excluded individuals with missing genotype or covariate data, leaving 787 children. We then rank-normalized the methylation data to remove outliers and controlled for covariates, potential batch effects and the influence of cell heterogeneity by regressing data points on sex, the first 10 ancestry principal components, bisulfite-converted DNA batch and blood cell proportions estimated using the Houseman method [33,34]. We then calculated residuals, which were used as the outcome variable in a linear regression model in PLINK [29] to calculate the relevant CpG‐SNP associations.

Finally, we excluded any mQTL acting in trans (i.e., any SNP associated with a CpG site more than 1 M base pairs away) and excluded any CpGs that have been flagged as potentially problematic (e.g., cross-hybridizing probes) according to a previous publication [35].

##### GOYA

We attempted to replicate mQTL from ALSPAC using genotype and cord blood DNA methylation data from the Genetics of Overweight Young Adults (GOYA) cohort, which is a subset of the Danish National Birth Cohort (DNBC) [36]. Genotype and cord blood DNA methylation data were available for 1000 children. We replicated the methods described above for ALSPAC by first excluding individuals with missing genotype or covariate data, leaving 889 children and also removing SNPs with missingness (>5%) using PLINK. As in ALSPAC, we rank-normalised the methylation data to remove outliers and adjusted for covariates, potential batch effects and the influence of cell heterogeneity by regressing data points on sex, the first ten ancestry principal components, DNA batch and blood cell proportions estimated using the Houseman method [33,34]. Residuals were then used as the outcome variable in a linear regression model in PLINK to calculate the relevant CpG‐SNP associations.

#### Expression quantitative trait loci as genetic risk variants

##### Genotype-Tissue Expression project

To identify expression quantitative trait loci (eQTL; SNPs associated with gene expression), we used the Genotype-Tissue Expression project (GTex) data, which is a database of eQTL generated using genotype and RNA sequencing gene expression data for 43 distinct tissue types from 175 individuals [37,38].

##### NESDA NTR conditional eQTL catalog

To explore the consistency of our findings, we also identified eQTL using a second database: the NESDA NTR Conditional eQTL Catalog. For this database, eQTL were identified using genotype and gene expression microarray data from blood samples from 4896 individuals across two Dutch biobanks. Conditional eQTL analysis was applied to distinguish between dependent and independent eQTL [39].

#### DNA methylation in children with orofacial clefts

##### Brazilian cohort

If genetic liability to nsCL/P is associated with DNA methylation at certain CpGs, we would expect these CpGs to be differentially methylated between nsCL/P cases and controls. To assess whether this was the case at nsCL/P-associated CpGs (identified through MR), we performed a look-up of results from a recently-published case-control EWAS [16]. This EWAS compared blood DNA methylation profiles in 67 nonfamilial, nsCL/P cases and 59 age- and sex-matched controls from a Brazilian population. The average age at sampling was 5.29 years for cases and 6.45 years for controls. DNA methylation was measured using the Illumina Infinium HumanMethylation450 BeadChip platform.

##### The Cleft Collective

To explore whether methylation at nsCL/P-associated CpGs differs by cleft subtype, we compared mean methylation values in blood and matched lip/palate tissue samples from 150 children from the UK enrolled in the Cleft Collective birth cohort study. Methylation data were generated for a separate study, as previously described[3]. Briefly, a sample of 150 believed to be nonsyndromic children was randomly selected and stratified by cleft subtype: 50 with CLO, 50 with CPO and 50 with CLP. These children have been classified as nonsyndromic because they have not been diagnosed as having any other anomaly, however, since the children are still very young, we cannot be completely sure of their nonsyndromic status. Blood and either lip or palate tissue samples were available for each of the 150 children in this study. The orofacial tissue type was dependent on the orofacial cleft subtype; therefore, lip samples were available for children with CLO and palate samples for children with CPO. Of the 50 children with CLP, 43 contributed a lip sample and seven contributed a palate sample. Genome-wide DNA methylation was measured using the Illumina Infinium HumanMethylation450 BeadChip platform and functional normalization was performed on the blood and tissue samples together. Of the original 300 samples, three blood and two lip samples failed quality control. Surrogate variables were generated using the sva package in R to capture variation in the methylation data associated with technical batch and cellular heterogeneity [40].

#### Analysis pipeline

##### Testing for mediation: MR of the effect of methylation on liability to nsCL/P

nsCL/P meta-GWAS summary statistics for 543,150 SNPs were LD-pruned (r^2^ < 0.001) to 17,090 independent SNPs. These independent SNPs were then merged with 127,215 mQTL from the ALSPAC mQTL database. After removing potentially problematic CpGs and CpGs acting in trans (which may increase the likelihood of horizontal pleiotropy), there were 7091 independent CpG‐SNP pairings for 6425 distinct CpGs. We then used the MR-base R package [41] to perform two-sample MR on all CpGs, using mQTL as the exposure variables and nsCL/P as the outcome. In initial analysis, CpGs with one mQTL were tested using the Wald test and CpGs with more than one mQTL were tested using the Inverse variance weighted (IVW) method. To account for possible residual LD between mQTL, CpGs with more than one mQTL, were retested adjusting for LD between the SNPs using a likelihood-based method [42]. Pair-wise SNP LD was computed using the Caucasian European (CEU) and British (GBR) populations in LDlink [43]. As a sensitivity analysis, we attempted to replicate the SNP-CpG associations with a Bonferroni-corrected MR p-value < 0.05 in GOYA, an independent sample, to further evaluate the evidence that the SNP-CpG associations of interest are genuine.

##### Testing for reverse causation: MR of the effect of genetic liability to nsCL/P on methylation

The MR analysis of methylation on nsCL/P used a single genetic instrument for the majority of CpGs, so it is difficult to discern the direction of effect. Therefore, we used MR-base to conduct the reverse two-sample MR to assess possibility B), reverse causation, that liability to nsCL/P causes downstream effects in methylation. Six genome-wide significant nsCL/P SNPs in Europeans [7] were used as the exposure and mQTL from ALSPAC were used as the outcome. The IVW method was used as the primary analysis.

##### Testing for linkage: joint-likelihood mapping to assess colocalization

We used the Joint likelihood mapping (JLIM) package in R ([jlim.R] [44] to test possibility C), liability to nsCL/P and methylation are driven by the same causal effect in each region of interest, in other words, the mQTL and nsCL/P risk variant are colocalized rather than simply being in LD. To distinguish between separate causal variants, we set the limit of genetic resolution in terms of r^2^ to 0.8. 1000 Genomes [45] CEU data were used as the reference dataset for LD. Most CpGs were associated with only one independent mQTL, so we were not able to distinguish mediation/vertical pleiotropy (Figure 1A) from horizontal pleiotropy (Figure 1D).

##### Comparison to gene expression

The previous steps identified CpGs that potentially mediate the effect of genetic variation on susceptibility to nsCL/P. Further evidence for a functional effect would be provided if mQTL also affected gene expression. Therefore, we looked up relevant SNPs in two eQTL databases (GTEx [38] and NESDA NTR Conditional eQTL Catalog [39]) and noted the estimated effect size and p-values for eQTL in various tissues.

##### Comparison to EWAS results

At identified CpGs, we looked-up the mean methylation values in nsCL/P cases and controls from the Brazilian EWAS study to determine if CpGs of interest from the MR were differentially methylated between nsCL/P cases and controls. Evidence for differential methylation would further support an etiological role in nsCL/P. We compared the direction of estimated effect and p-values obtained using the observational EWAS and MR approaches.

##### Tissue and cleft-subtype-specific variation

At identified CpGs, we used data from the Cleft Collective to explore whether methylation in blood was correlated with methylation at the site of the cleft (lip/palate), and whether mean methylation varied according to cleft subtype (CLO, CPO or CLP). One-way ANOVA was used to compare the mean methylation of subtypes, adjusting for sex and surrogate variables designed to capture technical batch and cell composition effects.

## Results

### Testing for mediation: MR of the effect of methylation on liability to nsCL/P

To identify CpGs where methylation may mediate genetic liability to nsCL/P, we used two sample MR with mQTL from the ALSPAC data as the exposure and liability to nsCL/P as the outcome (from the nsCL/P GWAS meta-analysis summary statistics). We found evidence for an effect of methylation on liability to nsCL/P at 26 CpGs after a multiple testing correction for the number of different CpGs tested (Bonferroni-corrected p-value < 0.05, corresponding to an uncorrected p-value < 7.8 × 10^-6^). Of these 26 CpGs, 20 were instrumented by single mQTL and six were instrumented by two mQTL each. When the six CpGs with two mQTL each were re-tested, taking into account LD between the SNPs, only one (cg02598441 at *LOC146880*) survived correction for multiple testing. These 21 mQTL were therefore taken forward to the reverse-causation step.

As a sensitivity analysis, we investigated all 21 of the ALSPAC mQTL in data from the GOYA cohort. 17 of the 21 CpG‐SNP pairings passed quality control and were present in the GOYA data, of which 16 replicated in the same direction with p < 0.05 (Supplementary Table 1).

### Testing for reverse causation: MR of the effect of genetic liability to nsCL/P on methylation

Next, we tested if the association between the mQTL and liability to nsCL/P arose because genetic liability to nsCL/P influences variation in methylation, by using two sample MR with genetic liability to nsCL/P as the exposure and methylation as the outcome. We found no strong evidence that genetic liability to nsCL/P influences variation in methylation at the 21 CpGs (Table 1). However, it should be noted that this step is very likely to be limited by statistical power.

**Table 1.** **Results of the forward (methylation → nonsyndromic cleft lip with or without cleft palate) and reverse (nonsyndromic cleft lip with or without cleft palate → methylation) Mendelian randomization and the colocalization analyses in Avon Longitudinal Study of Parents and Children.**

**SNP (allele 1/allele 2; annotated gene)**	**CpG (annotated gene)**	**Forward MR (effect size [standard error]; p-value)**	**Reverse MR (effect size [standard error]; p-value)**	**Colocalization (JLIM statistic; p-value by permutation)**
rs12057415 (T/C; n/a)	cg09549015 (*F3*)	-1.1 (0.2); 1.1 × 10^-6^	0.04 (0.07); 0.59	-8.5; 1

rs12057415 (T/C; n/a)	cg26112574 (n/a)	0.7 (0.1); 1.1 × 10^-6^	0.00 (0.05); 1.00	-16.7; 1

rs861020 (A/G; *IRF6*)	cg12766975 (*IRF6*)	1.1 (0.2); 1.1 × 10^-6^	0.00 (0.04); 0.99	-11.8; 1

rs861020 (A/G; *IRF6*)	cg09163369 (*C1orf107*)	-0.5 (0.1); 1.1 × 10^-6^	-0.04 (0.08); 0.59	-36.1; 1

rs861020 (A/G; *IRF6*)	cg23166289 (*C1orf107*)	-0.7 (0.2); 1.1 × 10^-6^	-0.01 (0.06); 0.92	-36.8; 1

rs861020 (A/G; *IRF6*)	cg05527609 (*C1orf107*)	0.9 (0.2); 1.1 × 10^-6^	-0.06 (0.06); 0.31	-2.1; 0.69

rs4422741 (C/T; n/a)	ch.8.2579072R (n/a)	0.7 (0.1); 2.1 × 10^-10^	0.11 (0.06); 0.08	-2.4; 0.91

rs4752028 (C/T; *SHTN1*)	cg00750430 (*SHTN1*)	1.2 (0.2); 8.7 × 10^-9^	0.13 (0.11); 0.25	-16.7; 1

rs4752028 (C/T; *SHTN1*)	cg03968911 (*SHTN1*)	-0.8 (0.1); 8.7 × 10^-9^	-0.11 (0.19); 0.58	-32.2; 1

rs4752028 (C/T; *SHTN1*)	cg11398452 (*VAX1*)	-0.8 (0.1); 8.7 × 10^-9^	-0.11 (0.19); 0.56	30.2; < 0.001

rs1258763 (C/T; n/a)	cg04870120 (n/a)	-1.2 (0.3); 1.3 × 10^-6^	0.04 (0.05); 0.38	-68.4; 1

rs1873147 (G/A; n/a)	cg04194852 (*TPM1*)	1.5 (0.3); 1.5 × 10^-8^	0.09 (0.10); 0.38	-13.1; 1

rs8076457 (T/C; *NTN1*)	cg18901140 (n/a)	-1.1 (0.2); 3.0 × 10^-7^	0.02 (0.07); 0.74	-34.6; 1

rs8076457 (T/C; *NTN1*)	cg19788727 (*NTN1*)	-0.9 (0.2); 3.0 × 10^-7^	0.03 (0.05); 0.51	-13.9; 1

rs8076457 (T/C; *NTN1*)	cg02481697 (*NTN1*)	-0.8 (0.2); 3.0 × 10^-7^	0.01 (0.05); 0.83	0.65; 0.01

rs8076457 (T/C; *NTN1*)	cg01862363 (*NTN1*)	-0.6 (0.1); 3.0 × 10^-7^	0.03 (0.09); 0.78	0.11; 0.016

rs8076457 (T/C; *NTN1*)	cg16107528 (*NTN1*)	-0.7 (0.1); 3.0 × 10^-7^	-0.01 (0.05); 0.98	4.3; < 0.001

rs1808191 (C/A; *PLEKHM1P1*)	cg14501219 (*LOC146880*)	-1.0 (0.2); 2.9 × 10^-6^	0.09 (0.05); 0.051	N/A^†^

rs1991401 (G/A; *CEP95*)	cg02598441 (*LOC146880*)	0.4 (0.1); 4.3 × 10^-7^	-0.04 (0.07); 0.59	N/A^‡^

rs1808191 (C/A; *PLEKHM1P1*)				

rs3746101 (T/G; *MKNK2*)	cg05254098 (*MKNK2*)	-1.0 (0.2); 5.0 × 10^-6^	-0.03 (0.05); 0.54	-37.2; 1

rs3746101 (T/G; *MKNK2*)	cg17068236 (*MKNK2*)	0.8 (0.2); 5.0 × 10^-6^	-0.02 (0.05); 0.58	-91.0; 1

^†^This region was too sparsely genotyped to apply the colocalization analysis.

^‡^This CpG had two mQTL, so we did not apply the colocalization analysis.

JLIM: Joint likelihood mapping; MR: Mendelian randomization; mQTL: Methylation quantitative trait loci; SNP: Single nucleotide polymorphism.

### Testing for linkage: joint-likelihood mapping to assess colocalization

We used JLIM, a colocalization method, to assess if there was evidence that methylation and liability to nsCL/P are driven by the same causal effect at each locus. Of the 20 CpGs instrumented by one mQTL each, we found evidence for colocalization at four CpGs (cg11398452, cg01862363, cg02481697 and cg16107528). With the addition of the CpG site associated with two mQTL (cg02598441), we found strongest evidence that methylation at five CpGs are putative mediators of genetic liability to nsCL/P at four different SNPs (Table 1).

Of these four SNPs, three were available and tested in the imputed GOYA data (rs807647, rs1808191 and rs4752028). Two of the SNPs (intergenic rs8076457 and rs1808191 near *PLEKHM1P1*) consistently replicated as mQTL in GOYA, with the third SNP rs4752028 replicating as an mQTL for two out of three CpG sites but not the CpG‐SNP pairing with evidence of colocalization (Supplementary Table 1).

### Comparison between methylation & gene expression

To assess further evidence for a functional effect of our identified mQTL on gene expression, we performed a look-up of the four identified SNPs in the GTex and NESDA NTR Conditional eQTL databases. We found strong evidence that rs4752028 at *SHTN1* is an eQTL for *SHTN1* in whole blood (Table 2). There was also strong evidence that both rs1808191 at *PLEKHM1P1* and rs1991401 at *CEP95/DDX5* are eQTL for six nearby genes, including *CEP95* (whole blood) and *DDX5* (whole blood), which were identified through both databases (Table 2). There was no available evidence in either catalogue that intergenic SNP rs8076457 is associated with gene expression (Table 2).

**Table 2.** **Associations with gene expression at identified SNPs in two expression quantitative trait loci databases.**

**SNP (annotated gene)**	**CpG (annotated gene)**	**GTex: Gene, tissue, effect size, p-value**	**eQTL: Expression quantitative trait loci; NESDA NTR Conditional eQTL Catalog: Gene, tissue, effect size, p-value**
rs4752028 (*SHTN1*)	cg11398452 (*VAX1*)	*SHTN1*, whole blood, 0.35, 1.4 × 10^-15^	*SHTN1*, whole blood, 0.68, 2.7 × 10^-159^

rs8076457 (*NTN1*)	cg01862363 (*NTN1*)	N/A	N/A

	cg02481697 (*NTN1*)		

	cg16107528 (*NTN1*)		

rs1808191 (*PLEKHM1P1*)	cg02598441 (*LOC146880*)	*RP13-104F24.3*, sun exposed skin, -0.31, 1.8 × 10^-15^ *SMURF2*, transformed fibroblasts, 0.28, 3.8 × 10^-13^ *has-mir-6080*, sun exposed skin, -0.29, 1.5 × 10^-11^ *PLEKHM1P*, sun exposed skin, -0.13, 3.2 × 10^-5^	N/A

rs1991401 (*CEP95*)		*DDX5*, whole blood, -0.30, 1.8 × 10^-19^ *CEP95*, whole blood, 0.14, 2.3 × 10^-7^ *MILR1*, whole blood, 0.24, 1.4 × 10^-5^	*DDX5*, whole blood, -0.22, 6.2 × 10^-108^ *CEP95*, whole blood, 0.13, 1.3 × 10^-16^

eQTL: Expression quantitative trait loci; NESDA: Netherlands study of depression and anxiety; NTR: Netherlands twin register; SNP: Single nucleotide polymorphism.

### Comparison to EWAS results

To provide further evidence for an association between methylation and nsCL/P at our identified CpGs, we compared the MR results (relating to liability to nsCL/P in the general population) to results from an nsCL/P case–control EWAS study. At cg02598441 (*LOC146880*), the direction of effect estimated in our first (forward) MR analysis was concordant with that in the EWAS study, with an EWAS p-value (2.4 × 10^-3^) that survived Bonferroni correction for five tests (Table 3). The direction of estimated effect was also concordant between studies at the three CpGs at *NTN1*, but the smallest EWAS p-value was 0.12. At cg11398452 (*VAX1*), the direction of estimated effect was discordant between our MR analysis and the Brazilian EWAS, with a small EWAS p-value (9.4 × 10^-3^; Table 3).

**Table 3.** **Comparison to methylation data in blood samples from children with an orofacial cleft.**

**SNP (annotated gene)**	**CpG (annotated gene)**	**Forward MR: Effect size^†^[standard error]; p-value)**	**methWAS nsCL/P EWAS: Effect size ^‡^[standard error]; p-value)**	**Correlation between blood and lip in the Cleft Collective: Correlation coefficient (95% C.I.)**	**Correlation between blood and palate in the Cleft Collective: Correlation coefficient (95% C.I.)**	**p-value for difference in mean blood DNA methylation between CLO, CLP and CPO in the Cleft Collective**
rs4752028 (*SHTN1*)	cg11398452 (*VAX1*)	-0.8 (0.1); 8.7 × 10^-9^	0.01 (0.05); 9.4 × 10^-3^	0.20 (-0.01, 0.41)	0.07 (-0.00, 0.14)	0.148

rs8076457 (*NTN1*)	cg01862363 (*NTN1*)	-0.8 (0.2); 3.0 × 10^-7^	-0.030 (0.037); 2.1 × 10^-1^	-0.04 (-0.24, 0.16)	-0.02 (-0.26, 0.22)	0.828

cg02481697 (*NTN1*)	-0.6 (0.1); 3.0 × 10^-7^	-0.023 (0.024); 1.7 × 10^-1^	0.29 (0.09, 0.49)	-0.06 (-0.35, 0.23)	0.286	

cg16107528 (*NTN1)*	-0.7 (0.1); 3.0 × 10^-7^	-0.019 (0.016); 1.2 × 10^-1^	0.34 (0.14, 0.54)	-0.11 (-0.37, 0.15)	0.646	

rs1808191 (*CEP95*) rs1991401 (*PLEKHM1P1*)	cg02598441 (*LOC146880*)	0.4 (0.1); 4.3 × 10^-7^	0.020 (0.007); 2.4 × 10^-3^	0.32 (0.12, 0.52)	0.13 (-0.15, 0.41)	0.548

rs1991401 (*PLEKHM1P1*)						

^†^Effect size for forward MR can be interpreted as the difference in risk of nonsyndromic CL/P per standard deviation increase in methylation beta value.

^‡^Effect size for the methWAS EWAS can be interpreted as the difference in mean methylation beta value in participants with nonsyndromic CL/P compared with controls.

CLO: Cleft lip only; CLP: Cleft lip with cleft palate CPO: Cleft palate only; CL/P: Cleft lip with or without cleft palate; EWAS: Epigenome-wide association study; SNP: Single nucleotide polymorphism.

### Tissue & cleft-subtype-specific variation

We assessed correlations in methylation at our identified CpGs between different tissues (blood and lip/palate) and cleft subtypes (CPO, CLP, CLO) to explore tissue- and subtype-specific variation. At most of the five identified CpGs, there was evidence of weak correlation (correlation coefficients ranging -0.11 to 0.32) between methylation in blood, lip and palate tissues, particularly between blood and lip tissue. We found weak evidence that mean methylation values in any of the three tissues differed between cleft subtypes. However, the analysis was likely underpowered to give precise correlation estimates (Table 3).

## Discussion

In this study, we employed a framework that aims to identify putative mediators of genetic influences on nsCL/P via DNA methylation. We found five CpG sites, in three independent regions (*VAX1, LOC146880, NTN1*), where bidirectional MR and colocalization analyses suggested that the same variant affects both methylation and nsCL/P or where evidence was found that two independent variants affect both nsCL/P and methylation (Figure 1).

We found lower methylation at the CpG at *VAX1* (cg11398452) in association with the nsCL/P risk allele C of the SNP rs4752028 at *SHTN1*. This SNP is strongly associated with lower expression of *SHTN1* according to two eQTL databases. *VAX1* is a homeobox containing gene that has been shown to be expressed in the developing brain [46,47] and SNPs in *VAX1* have been shown to be associated with nsCL/P in multiple independent GWAS across distinct populations [4,8,9,48,49]. *VAX1* knock-out mice have been shown to develop cleft palate, suggesting *VAX1* has a potentially important role in nsCL/P etiology [4]. *SHTN1*, sometimes known as *KIAA1598*, codes for the protein shootin1 that is involved in neuronal polarization [50] and has also been reported to be relevant to the etiology of nsCL/P in several studies [9,51,52]. It is difficult to distinguish the more significant locus between the *VAX1* and *SHTN1* genes because of their close proximity and similar expression profiles in mice [9,47,52,53]. We did not replicate the association between methylation at cg11398452 and rs4752028 in the GOYA data, but we did find that the SNP was strongly associated with methylation at nearby probes. Similarly, the direction of association between cg11398452 methylation and nsCL/P was the opposite in our MR study, compared with a previously published observational EWAS [16].

We found higher methylation at the CpG at *LOC146880* (cg02598441) in association with the G allele of rs1991401 in *DDX5* and the C allele of rs1808191 in *PLEKHM1P*. rs1991401 in *DDX5* was associated with reduced expression of *DDX5* and increased expression of *CEP95* and *MILR1* while rs1808191 in *PLEKHM1P* was associated with increased expression of *SMURF2* but decreased expression of *PLEKHM1P*, *RP13-104F24.3* and *has-mir-6080*. However, there was weak evidence that the SNPs affected expression of the same genes. *DDX5* is involved in RNA helicase processes that are highly relevant to important cellular processes while *PLEKHM1P* and *LOC146880* are pseudogenes [47]. There is no robust evidence from previous literature to support an association between genetic variation in these genes and nsCL/P. The SNP in *PLEKHM1P* replicated as an mQTL in the GOYA dataset but there were insufficient data to test the SNP in *DDX5*.

We found lower methylation at three CpGs at *NTN1* in association with the nsCL/P risk allele T of the SNP rs8076457, an intergenic SNP close to *NTN1*. rs8076457, the mQTL for cg08162363, cg02481697 and cg16107528, did not robustly associate with gene expression levels in two datasets. The function of *NTN1* is still largely unknown, but is thought to be involved in cell migration during development [47]. *NTN1* has been previously discussed as a strong candidate gene for nsCL/P [13]; *NTN1* may affect liability to nsCL/P via epistatic interactions, there is some evidence that *NTN1* knock-out mice show consistency with the cleft palate phenotype and *NTN1* expression is localized to the palate [13,54]. rs8076457 replicated as an mQTL across all relevant CpGs in the GOYA dataset.

Previous work has identified many functional possibilities for genetic risk variants for nsCL/P [15,54,55] but this study adds to the current evidence for DNA methylation playing a role in the etiology of nsCL/P. Additional strengths of this study include the integration of multiple data sources, for example ALSPAC, which provided access to detailed phenotype, genotype and epigenetic data. The nsCL/P GWAS summary statistics allowed a comprehensive genome-wide analysis in a large dataset. The Brazilian cohort EWAS results allowed a comparison of the influence of methylation on nsCL/P according to observational and MR studies. The use of the GOYA replication cohort, allowed triangulation of evidence for mQTL across different studies. Finally, the Cleft Collective data allowed us to compare genome-wide DNA methylation in different tissues and subtypes of cleft.

There are, several limitations to this study. First, methylation and expression in the studied tissues (postnatal cord blood, whole blood, lip and palate tissue) are unlikely to accurately reflect that in the developing orofacial tissue where epigenetic processes could feasibly influence susceptibility to nsCL/P. Since it is not possible to study DNA methylation in the developing orofacial region of affected individuals, we explored correlations between blood and lip/palate as a best available alternative. We found weak correlation between DNA methylation at our putative causal regions in blood and lip/palate samples from individuals in the Cleft Collective. A previous study found high blood-lip/palate correlation at CpGs identified as differentially methylated between nsCL/P cases and controls [16]. Correlations in methylation signatures across tissues in the same individuals provide support for effects that are not specific to certain tissues. For example, a recent study showed strong correlation of cis- eQTL and mQTL effects between two tissues (brain and blood) [56]. However, without data on the relevant tissue, it remains possible that the methylation and expression signatures of blood, lip and palate could be poorly representative of those in the developing orofacial tissues. Second, CLO and CLP cases were analyzed together as one group in the GWAS, MR analyses and the previously published EWAS. Increasingly, evidence suggests that these subtypes are molecularly and etiologically distinct and should be analyzed separately [2,3], but we were limited by the data available from previous studies. Although we found no evidence of differential methylation between subtypes at our five identified CpGs, there may be other loci where methylation mediates genetic influences on more specific cleft subtypes. Third, although efforts were made to select only nonsyndromic cases for the Cleft Collective analysis, we cannot guarantee that no syndromic cases were included, and children with syndromes may have very different methylation profiles. Fourth, a major limitation of this study is that some of the steps, particularly the reverse MR (testing the effect of liability to nsCL/P on methylation), are likely to be statistically underpowered. Liability to nsCL/P has been previously shown to have a causal affect on facial morphology [57] suggesting that it could also affect methylation. Fifth, as the majority of mQTL were instrumented by just a single genetic variant, we were unable to distinguish between mediation and horizontal pleiotropy and therefore proposed mediation is putative. Finally, our comparisons with other cohorts may be affected by technical differences, tissue differences (cord blood vs whole blood), ancestral differences between cohorts, age of participants (newborns vs children over 6 years old, a lack of statistical power giving rise to spurious associations or the enrichment of GOYA for overweight and obese mothers, which may introduce selection bias. Indeed, although mQTL were largely concordant between ALSPAC and GOYA, the mQTL and CpG site (in *SHTN1*) found to colocalize with liability to nsCL/P in ALSPAC did not replicate in GOYA. Similarly, the CpG site cg11398452, close to *SHTN1*, was directionally discordant between our analyses and the results of a Brazilian EWAS.

## Conclusion

We identified three putative loci where DNA methylation may mediate genetic susceptibility to nsCL/P with stronger conclusions limited by the use of blood as a proxy for developing orofacial tissues. Future work determining the function of these genes and the epigenetic modulation of their expression relevant to prenatal orofacial development could provide important etiological insights. One possibility, warranting further investigation, is that identified DNA methylation differences are related to environmental exposures.

## Future perspective

Increase in the availability and size of integrated DNA methylation, gene expression and genotype datasets will enable comprehensive evaluation of the role of epigenetic processes in disease etiology. In particular for congenital traits like nsCL/P, the unavailability of the most ‘relevant’ embryonic tissue is always likely to be a limitation for human studies. However, large-scale efforts to identify genetic variants that influence epigenetic processes in a tissue-independent manner will be invaluable to MR studies that will help mitigate this concern. Relating nsCL/P-associated epigenetic processes to environmental exposures that are risk factors for nsCL/P, may lead to the development of targeted interventions.

**Summary points**

- Nonsyndromic cleft lip with or without cleft palate (nsCL/P) is a complex trait with genetic and environmental risk factors.
- Around 40 distinct genetic risk loci have been identified for nsCL/P, but many reside in nonprotein-coding regions with an unclear function.
- We hypothesize that epigenetic processes may play an important role in the etiology of nsCL/P.
- To explore whether nsCL/P genetic risk variants influence liability to nsCL/P through gene regulation pathways involving DNA methylation, we combined data from multiple sources and used an analysis framework involving Mendelian randomization and joint likelihood mapping.
- We find putative evidence that genetic variation in *VAX1, LOC146880* and *NTN1* may increase liability to nsCL/P via changes in DNA methylation.
- Methylation in blood, lip and palate tissue may not be strongly representative of that in the developing orofacial tissues, which limits stronger conclusions.
- The study highlights the potential role for DNA methylation in the etiology of nsCL/P.
- Future work exploring the influence of prenatal environmental factors on DNA methylation related to nsCL/P is warranted.

## References

[1] Mossey PA, Little J, Munger RG, Dixon MJ, Shaw WC (2009). Cleft lip and palate. *Lancet*.

[2] Leslie EJ, Carlson JC, Shaffer JR (2017). Genome-wide meta-analyses of nonsyndromic orofacial clefts identify novel associations between FOXE1 and all orofacial clefts, and TP63 and cleft lip with or without cleft palate. *Hum. Genet.*.

[3] Sharp GC, Ho K, Davies A (2017). Distinct DNA methylation profiles in subtypes of orofacial cleft. *Clin. Epigen.*.

[4] Dixon MJ, Marazita ML, Beaty TH, Murray JC (2011). Cleft lip and palate: understanding genetic and environmental influences. *Nat. Rev. Genet.*.

[5] Setó-Salvia N, Stanier P (2014). Genetics of cleft lip and/or cleft palate: association with other common anomalies. *Eur. J. Med. Genet.*.

[6] Smith GD (2011). Epidemiology, epigenetics and the ‘gloomy prospect’: embracing randomness in population health research and practice. *Int. J. Epidemiol.*.

[7] Ludwig KU, Mangold E, Herms S (2012). Genome-wide meta-analyses of nonsyndromic cleft lip with or without cleft palate identify six new risk loci. *Nat. Genet.*.

[8] Mangold E, Ludwig KU, Birnbaum S (2010). Genome-wide association study identifies two susceptibility loci for nonsyndromic cleft lip with or without cleft palate. *Nat. Genet.*.

[9] Nikopensius T, Birnbaum S, Ludwig KU (2010). Susceptibility locus for non-syndromic cleft lip with or without cleft palate on chromosome 10q25 confers risk in Estonian patients. *Eur. J. Oral. Sci.*.

[10] Birnbaum S, Ludwig KU, Reutter H (2009). Key susceptibility locus for nonsyndromic cleft lip with or without cleft palate on chromosome 8q24. *Nat. Genet.*.

[11] Yu Y, Zuo X, He M (2017). Genome-wide analyses of non-syndromic cleft lip with palate identify 14 novel loci and genetic heterogeneity. *Nat. Commun.*.

[12] Sun Y, Huang Y, Yin A (2015). Genome-wide association study identifies a new susceptibility locus for cleft lip with or without a cleft palate. *Nat. Commun.*.

[13] Beaty T, Taub M, Scott A (2013). Confirming genes influencing risk to cleft lip with/without cleft palate in a case–parent trio study. *Hum. Genet.*.

[14] Beaty TH, Murray JC, Marazita ML (2010). A genome-wide association study of cleft lip with and without cleft palate identifies risk variants near *MAFB* and *ABCA4*. *Nat. Genet.*.

[15] Uslu VV, Petretich M, Ruf S (2014). Long-range enhancers regulating *Myc* expression are required for normal facial morphogenesis. *Nat. Genet.*.

[16] Alvizi L, Ke X, Brito LA (2017). Differential methylation is associated with non-syndromic cleft lip and palate and contributes to penetrance effects. *Sci. Rep.*.

[17] Sharp GC, Stergiakouli E, Sandy J, Relton C (2017). Epigenetics and orofacial clefts: a brief introduction. *Cleft Palate Craniofac. J.*.

[18] Juriloff DM, Harris MJ, Mager DL, Gagnier L (2014). Epigenetic mechanism causes Wnt9b deficiency and nonsyndromic cleft lip and palate in the A/WySn mouse strain. *Birth Defects Res. A. Clin. Mol. Teratol.*.

[19] Plamondon JA, Harris MJ, Mager DL, Gagnier L, Juriloff DM (2011). The *clf2* gene has an epigenetic role in the multifactorial etiology of cleft lip and palate in the A/WySn mouse strain. *Birth Defects Res. A Clin. Mol. Teratol.*.

[20] Richardson TG, Haycock PC, Zheng J (2017). Systematic Mendelian randomization framework elucidates hundreds of genetic loci which may influence disease through changes in DNA methylation levels. *bioRxiv*.

[21] Richardson TG, Zheng J, Smith GD (2017). Mendelian randomization analysis identifies CpG sites as putative mediators for genetic influences on cardiovascular disease risk. *Am. J. Hum. Genet.*.

[22] Gaunt TR, Shihab HA, Hemani G (2016). Systematic identification of genetic influences on methylation across the human life course. *Genome Biol.*.

[23] Davey Smith G, Ebrahim S (2003). ‘Mendelian randomization’: can genetic epidemiology contribute to understanding environmental determinants of disease?. *Int. J. Epidemiol.*.

[24] Bowden J, Davey Smith G, Burgess S (2015). Mendelian randomization with invalid instruments: effect estimation and bias detection through Egger regression. *Int. J. Epidemiol.*.

[25] Bowden J, Davey Smith G, Haycock PC, Burgess S (2016). Consistent estimation in Mendelian randomization with some invalid instruments using a weighted median estimator. *Genet. Epidemiol.*.

[26] Mailman MD, Feolo M, Jin Y (2007). The NCBI dbGaP database of genotypes and phenotypes. *Nat. Genet.*.

[27] Spielman RS, Mcginnis RE, Ewens WJ (1993). Transmission test for linkage disequilibrium: the insulin gene region and insulin-dependent diabetes mellitus (IDDM). *Am. J. Hum. Genet.*.

[28] Willer CJ, Li Y, Abecasis GR (2010). METAL: fast and efficient meta-analysis of genomewide association scans. *Bioinformatics*.

[29] Purcell S, Neale B, Todd-Brown K (2007). PLINK: a tool set for whole-genome association and population-based linkage analyses. *Am. J. Hum. Genet.*.

[30] Golding P, Jones and the Alspac Study Team (2001). ALSPAC – the avon longitudinal study of parents and children. *Paediatr. Perinat. Epidemiol.*.

[31] Boyd A, Golding J, Macleod J (2012). Cohort profile: the ‘children of the 90s’ – the index offspring of the Avon longitudinal study of parents and children. *Int. J. Epidemiol.*.

[32] Relton CL, Gaunt T, Mcardle W (2015). Data resource profile: accessible resource for integrated epigenomic studies (aries). *Int. J. Epidemiol.*.

[33] Houseman EA, Accomando WP, Koestler DC (2012). DNA methylation arrays as surrogate measures of cell mixture distribution. *BMC Bioinform.*.

[34] Reinius LE, Acevedo N, Joerink M (2012). Differential DNA methylation in purified human blood cells: implications for cell lineage and studies on disease susceptibility. *PLoS ONE*.

[35] Zhou W, Laird PW, Shen H (2017). Comprehensive characterization, annotation and innovative use of Infinium DNA methylation BeadChip probes. *Nucleic Acids Res.*.

[36] Paternoster L, Evans DM, Nohr EA (2011). Genome-wide population-based association study of extremely overweight young adults – the GOYA study. *PLoS ONE*.

[37] GTex consortium (2015). The Genotype-Tissue Expression (GTEx) pilot analysis: Multitissue gene regulation in humans. *Science*.

[38] Lonsdale J, Thomas J, Salvatore M (2013). The genotype-tissue expression (GTEx) project. *Nat. Genet.*.

[39] Jansen R, Hottenga J-J, Nivard MG (2017). Conditional eQTL analysis reveals allelic heterogeneity of gene expression. *Hum. Mol. Genet.*.

[40] Leek JT, Johnson WE, Parker HS, Jaffe AE, Storey JD (2012). The sva package for removing batch effects and other unwanted variation in high-throughput experiments. *Bioinformatics*.

[41] Hemani G, Zheng J, Elsworth B (2018). The MR-Base platform supports systematic causal inference across the human phenome. *eLife*.

[42] Burgess S, Dudbridge F, Thompson SG (2016). Combining information on multiple instrumental variables in Mendelian randomization: comparison of allele score and summarized data methods. *Stat. Med.*.

[43] Machiela MJ, Chanock SJ (2015). LDlink: a web-based application for exploring population-specific haplotype structure and linking correlated alleles of possible functional variants. *Bioinformatics*.

[44] Chun S, Casparino A, Patsopoulos NA (2017). Limited statistical evidence for shared genetic effects of eQTLs and autoimmune-disease-associated loci in three major immune-cell types. *Nat. Genet.*.

[45] 1000 Genomes Project consortium (2015). A global reference for human genetic variation. *Nature*.

[46] Hallonet M, Hollemann T, Pieler T, Gruss P (1999). *Vax1*, a novel homeobox-containing gene, directs development of the basal forebrain and visual system. *Genes Dev.*.

[47] Pruitt KD, Maglott DR (2001). RefSeq and LocusLink: NCBI gene-centered resources. *Nucleic Acids Res.*.

[48] Butali A, Suzuki S, Cooper ME (2013). Replication of genome wide association identified candidate genes confirm the role of common and rare variants in *PAX7* and *VAX1* in the etiology of nonsyndromic CL (P). *Am. J. Med. Genet. Part A*.

[49] De Aquino SN, Messetti AC, Bagordakis E (2013). Polymorphisms in *FGF12*, *VCL*, *CX43* and *VAX1* in Brazilian patients with nonsyndromic cleft lip with or without cleft palate. *BMC Med. Genet.*.

[50] Toriyama M, Shimada T, Kim KB (2006). Shootin1: a protein involved in the organization of an asymmetric signal for neuronal polarization. *J. Cell Biol.*.

[51] Wang Y, Sun Y, Huang Y (2016). Validation of a genome-wide association study implied that SHTIN1 may involve in the pathogenesis of NSCL/P in Chinese population. *Sci. Rep.*.

[52] Mostowska A, Hozyasz KK, Wojcicka K, Biedziak B, Jagodzinski PP (2012). Polymorphic variants at 10q25. 3 and 17q22 loci and the risk of non-syndromic cleft lip and palate in the polish population. *Birth Defects Res. A Clin. Mol. Teratol.*.

[53] Carlson JC, Taub MA, Feingold E (2017). Identifying genetic sources of phenotypic heterogeneity in orofacial clefts by targeted sequencing. *Birth Defects Res.*.

[54] Leslie EJ, Taub MA, Liu H (2015). Identification of functional variants for cleft lip with or without cleft palate in or near *PAX7*, *FGFR2*, and *NOG* by targeted sequencing of GWAS loci. *Am. J. Hum. Genet.*.

[55] Leslie EJ, Murray JC (2013). Evaluating rare coding variants as contributing causes to non-syndromic cleft lip and palate. *Clin. Genet.*.

[56] Qi T, Wu Y, Zeng J (2018). Identifying gene targets for brain-related traits using transcriptomic and methylomic data from blood. *Nat. Commun.*.

[57] Howe LJ, Lee MK, Sharp GC (2018). Investigating the shared genetics of non-syndromic cleft lip/palate and facial morphology. *PLoS Genet.*.

