# Supplementary material for: Evidence for DNA methylation mediating genetic liability to non-syndromic cleft lip/palate
Source: Epigenomics. 2019 Jan 14;11(2):133–45. doi: 10.2217/epi-2018-0091 (PMC6462847; doi:10.2217/epi-2018-0091)
Supplement: Supplementary file 1 [file epi-11-133-s1.docx]

# Supplementary Material

## nsCL/P GWAS methods

The transmission disequilibrium test (TDT) ^1^ evaluates the frequency with which parental alleles are transmitted to affected offspring and is a family based association test of genetic linkage in the presence of genetic association. The TDT was run on 638 parent-offspring trios and 178 parent-offspring duos of European, descent, publicly available from dbGAP, to determine genome-wide genetic variation associated with nsCL/P. GWAS genotypes and phenotypes available at dbGaP (<https://www.ncbi.nih/gov/gap>; accession number phs000094.v1.p1).

The Bonn-II study ^2^ summary statistics from a case-control GWAS of 399 nsCL/P cases and 1,318 controls were meta-analysed using a fixed effect inverse-variance weighted method, in terms of effect size and standard error, with the TDT GWAS summary statistics using METAL ^3^ based on a previously described protocol for combining TDT and case-control studies ^4^. The final sample including 1215 cases and 2772 controls.

### Avon Longitudinal Study of Parents and Children (ALSPAC)

To identify mQTL (SNPs associated with DNA methylation), we used data from the Avon Longitudinal Study of Parents and Children (ALSPAC). ALSPAC is a longitudinal study that recruited pregnant women living in the former county of Avon (UK) with expected delivery dates between 1 April 1991 and 31 December 1992^5 6^. Written, informed consent was obtained for all participants. Ethics approval for the study was obtained from the ALSPAC Ethics and Law Committee and the Local Research Ethics Committee. The study website contains details of all available data through a searchable data dictionary (<http://www.bristol.ac.uk/alspac/researchers/dataaccess/datadictionary/)>. In addition to collecting detailed questionnaire and clinic data for the whole cohort, the study has generated genome-wide DNA methylation and genotype data for subsets.

As part of the Accessible Resource for Integrated Epigenomic Studies (ARIES) project ^7^, genome-wide DNA methylation data were generated for 1018 ALSPAC mother-child pairs across five time-points. For the purposes of the current study, we used data generated using offspring cord blood samples collected at birth. Generation of these data are described in detail elsewhere. Briefly, DNA samples were bisulfite treated and DNA methylation was quantified using the Illumina Infinium HumanMethylation450K BeadChip assay, which measures DNA methylation at over 480,000 CpG sites across the genome. After quality control and functional normalisation using the R package meffil ^8^, data were reported as methylation beta values, ranging from 0 (completely unmethylated) to 1 (completely methylated).

Genotype data were available for all 1018 children in ARIES, generated using the Illumina HumanHap550 quad genome-wide SNP genotyping platform. Individuals were excluded from further analysis based on having incorrect gender assignments, minimal or excessive heterozygosity (0.345 for the Sanger data and 0.330 for the LabCorp data), disproportionate levels of individual missingness (>3%), evidence of cryptic relatedness (>10% IBD) and being of non-European ancestry (as detected by a multidimensional scaling analysis seeded with HapMap 2 individuals.

ALSPAC methylation and genotype data have previously been used to generate a database of mQTL (<http://www.mqtldb.org/)> ^9^. The database contains summary statistics for all mQTL with a P-value <1*10-5 for the association between SNP and CpG. For part of our study, we required specific CpG-SNP associations that were unavailable from mQTLdb.org. Therefore, for required CpGs, we replicated the methods in the original study: we excluded individuals with missing genotype or covariate data, leaving 787 children. We then rank-normalised the methylation data to remove outliers and then controlled for covariates, potential batch effects and the influence of cell heterogeneity by regressing data points on sex, the first 10 ancestry principal components, bisulfite-converted DNA batch and blood cell proportions ^10 11^estimated using the Houseman method. The residuals were then used as the outcome variable in a linear regression model in PLINK^12^ to calculate the relevant CpG-SNP associations.

### Genetics of Overweight Young Adults (GOYA)

The Genetics of Overweight Young Adults (GOYA) study is described previously by Paternoster et al ^13^. It is based on the Danish National Birth Cohort that included 92,000 pregnant women and their pregnancies during 1996-2002. Of 67,853 women who had given birth to a live born infant, had provided a blood sample during pregnancy and had BMI information available, 3.6% of these women with the largest residuals from the regression of BMI on age and parity (all entered as continuous variables) were selected for GOYA. The BMI for these 2451 women ranged from 32.6 to 64.4. From the remaining cohort a random sample of similar size (2450) was also selected. DNA methylation data were generated for the offspring of 1000 mothers in the GOYA study. I.e. “cases” had mothers with a BMI>32 and “controls” were sampled from the normal BMI distribution (can include mothers with a BMI>32).

Methylation data were generated at the University of Bristol as described above for ALSPAC. Data were QCd and normalised using the meffil R package ^8^. Genome-wide genotyping on the Illumina 610k quad chip was carried out at the Centre National de Genotypage, Evry, France. Individuals were excluded from further analysis based on having incorrect gender assignments, minimal or excessive heterozygosity (>35% or <30.2%), disproportionate levels of individual missingness (>5%), relatedness and being of non-European ancestry (as detected by a multidimensional scaling analysis seeded with HapMap 2 individuals.

## Replication of mQTL results

**Supplementary Table 1: mQTL replication**

| **SNP (allele 1/allele 2; annotated gene)** | **CpG (annotated gene)** | **ALSPAC mQTL:** *Effect size^1^, P-value* | **GOYA mQTL:** *Effect size, P-value* | **Replicate with GOYA P<0.05** |
| --- | --- | --- | --- | --- |
| rs12057415 (T/C; n/a) | cg09549015 (*F3*) | 0.26, 9.4*10^-9^ | 0.008, 1.7*10^-17^ | Yes |
| rs12057415 (T/C; n/a) | cg26112574 (n/a) | -0.39, 1.2*10^-19^ | -0.028, 2.7*10^-43^ | Yes |
| rs861020 (A/G; *IRF6*) | cg12766975 (*IRF6*) | 0.29, 7.8*10^-9^ | 0.031, 2.7*10^-20^ | Yes |
| rs861020 (A/G; *IRF6*) | cg09163369 (*C1orf107*) | -0.60, 6.0*10^-24^ | -0.032, 9.3*10^-37^ | Yes |
| rs861020 (A/G; *IRF6*) | cg23166289 (*C1orf107*) | -0.44, 1.7*10^-15^ | -0.027, 1.1*10^-21^ | Yes |
| rs861020 (A/G; *IRF6*) | cg05527609 (*C1orf107*) | 0.34, 2.6*10^-9^ | -0.007, 6.2*10^-97^ | Yes |
| rs4422741 (C/T; n/a) | ch.8.2579072R (n/a) | 0.75, 1.3*10^-49^ | 0.030, 2.3*10^-17^ | Yes |
| rs4752028 (C/T; *SHTN1*) | cg00750430 (*KIAA1598*) | 0.34, 3.5*10^-11^ | 0.017, 2.1*10^-17^ | Yes |
| rs4752028 (C/T; *SHTN1*) | cg03968911 (*KIAA1598*) | -0.52, 2.7*10^-24^ | -0.049, 4.5*10^-45^ | Yes |
| rs4752028 (C/T; *SHTN1*) | cg11398452 (*VAX1*) | -0.51, 7.6*10^-27^ | -0.000, 0.27 | No |
| rs1258763 (C/T; n/a) | cg04870120 (n/a) | 0.25, 1.6*10^-9^ | N/A (didn’t have SNP) | N/A |
| rs1873147 (G/A; n/a) | cg04194852 (*TPM1*) | 0.24, 4.3*10^-8^ | 0.001, 1.1*10^-20^ | Yes |
| rs8076457 (T/C; *NTN1*) | cg18901140 (n/a) | -0.28, 3.2*10^-8^ | -0.018, 2.2*10^-7^ | Yes |
| rs8076457 (T/C; *NTN1*) | cg19788727 (*NTN1*) | -0.36, 8.6*10^-13^ | -0.016, 5.0*10^-15^ | Yes |
| rs8076457 (T/C; *NTN1*) | cg02481697 (*NTN1*) | -0.41, 1.1*10^-15^ | -0.061, 2.2*10^-22^ | Yes |
| rs8076457 (T/C; *NTN1*) | cg01862363 (*NTN1*) | -0.51, 1.6*10^-24^ | -0.085, 6.6*10^-30^ | Yes |
| rs8076457 (T/C; *NTN1*) | cg16107528 (*NTN1*) | -0.49, 1.9*10^-26^ | -0.039, 6.8*10^-27^ | Yes |
| rs1808191 (C/A; *PLEKHM1P1*) | cg14501219 (*LOC146880*) | -0.33, 1.5*10^-9^ | -0.014, 1.2*10^-19^ | Yes |
| rs1991401 (G/A; *CEP95*) | cg02598441 (*LOC146880*) | 0.25, 2.2*10^-8^ | N/A (didn’t have SNP) | N/A |
| rs1808191 (C/A; *PLEKHM1P1*) |  | 0.83, 3.6*10^-66^ | 0.021, 8.2*10^-112^ | Yes |
| rs3746101 (T/G; *MKNK2*) | cg05254098 (*MKNK2*) | -0.46, 5.3*10^-8^ | N/A (didn’t have SNP) | N/A |
| rs3746101 (T/G; *MKNK2*) | cg17068236 (*MKNK2*) | 0.57, 8.7*10^-11^ | N/A (didn’t have SNP) | N/A |

^1^ ALSPAC regression coefficients are on rank-normalised data

**REFERENCES**

1. Spielman RS, McGinnis RE, Ewens WJ. Transmission test for linkage disequilibrium: the insulin gene region and insulin-dependent diabetes mellitus (IDDM). *American journal of human genetics* 1993;52(3):506.

2. Mangold E, Ludwig KU, Birnbaum S, et al. Genome-wide association study identifies two susceptibility loci for nonsyndromic cleft lip with or without cleft palate. *Nature genetics* 2010;42(1):24-26.

3. Willer CJ, Li Y, Abecasis GR. METAL: fast and efficient meta-analysis of genomewide association scans. *Bioinformatics* 2010;26(17):2190-91.

4. Kazeem G, Farrall M. Integrating case‐control and TDT studies. *Annals of human genetics* 2005;69(3):329-35.

5. Golding P, Jones and the ALSPAC Study Team. ALSPAC–the avon longitudinal study of parents and children. *Paediatric and perinatal epidemiology* 2001;15(1):74-87.

6. Boyd A, Golding J, Macleod J, et al. Cohort profile: the ‘children of the 90s’—the index offspring of the Avon Longitudinal Study of Parents and Children. *International journal of epidemiology* 2012:dys064.

7. Relton CL, Gaunt T, McArdle W, et al. Data resource profile: accessible resource for integrated epigenomic studies (aries). *International journal of epidemiology* 2015;44(4):1181-90.

8. Min J, Hemani G, Smith GD, et al. Meffil: efficient normalisation and analysis of very large DNA methylation samples. *bioRxiv* 2017:125963.

9. Gaunt TR, Shihab HA, Hemani G, et al. Systematic identification of genetic influences on methylation across the human life course. *Genome biology* 2016;17(1):61.

10. Houseman EA, Accomando WP, Koestler DC, et al. DNA methylation arrays as surrogate measures of cell mixture distribution. *BMC bioinformatics* 2012;13(1):86.

11. Reinius LE, Acevedo N, Joerink M, et al. Differential DNA methylation in purified human blood cells: implications for cell lineage and studies on disease susceptibility. *PloS one* 2012;7(7):e41361.

12. Purcell S, Neale B, Todd-Brown K, et al. PLINK: a tool set for whole-genome association and population-based linkage analyses. *The American Journal of Human Genetics* 2007;81(3):559-75.

13. Paternoster L, Evans DM, Nohr EA, et al. Genome-wide population-based association study of extremely overweight young adults–the GOYA study. *PloS one* 2011;6(9):e24303.
